# Banana in a Condom: An Unusual Cause of Small Bowel Obstruction

**DOI:** 10.7759/cureus.34089

**Published:** 2023-01-23

**Authors:** Rosarie A Tudas, Karan Rao, Luis J Garcia, Yashant Aswani

**Affiliations:** 1 Radiology, University of Iowa Roy J. and Lucille A. Carver College of Medicine, Iowa City, USA; 2 Radiology, University of Iowa Hospitals and Clinics, Iowa City, USA; 3 Surgery, University of Iowa Hospitals and Clinics, Iowa City, USA

**Keywords:** small-bowel obstruction, ingested large foreign body, swallowed foreign body, foreign body ingestion in adults, condom ileus

## Abstract

An otherwise healthy, 34-year-old man presented to the emergency department with abdominal pain, nausea, and vomiting, which began a day after he ingested a banana-stuffed condom. A contrast-enhanced computed tomography (CT) abdomen and pelvis revealed a dilated small bowel with a transition point, findings consistent with small bowel obstruction. Abdominopelvic ascites was also noted on imaging, which was concerning for bowel distress. He was taken to the operating room for an exploratory laparotomy, which revealed a high-grade obstruction by a foreign body, a banana stuffed in a condom, in the mid-jejunum. An enterotomy was performed to relieve the obstruction. The postoperative course was uneventful, and he was discharged three days later.

## Introduction

Small bowel obstruction refers to the failure of the passage of intestinal contents through the bowel; causes can be functional or mechanical in nature [1]. The former, also known as ileus, results from dysfunctional peristalsis secondary to damage or insult to the intestinal smooth muscles or nerves. In contrast, mechanical obstruction occurs because of extraluminal compression of the bowel (e.g., adhesions, tumors, strictures) or intraluminal blockage (e.g., foreign body, intraluminal lesions) [2]. Small bowel obstruction is an unusual complication resulting from the ingestion of drug-filled condoms to smuggle illicit drugs. Here, we report a case in which a banana stuffed in a condom was ingested, resulting in small bowel obstruction.

## Case presentation

An otherwise healthy man with a history of depression presented to the emergency department with abdominal pain, nausea, and vomiting. His symptoms began several hours prior to the presentation and consisted of progressively worsening, non-radiating, generalized abdominal pain, accompanied by nausea and non-bloody emesis. He denied any identifiable aggravating or relieving factors. He had been unable to tolerate any food or fluids but continued to pass flatus, though decreased from his baseline. His last bowel movement was on the day prior to the onset of symptoms. He admitted to having swallowed a banana stuffed in a condom amidst a “hormonal rage” approximately 24 hours prior to his presentation. He denied chest pain, hematemesis, or hematochezia. He denied intent of self-harm or prior history of foreign body ingestion. He had no history of abdominal surgery or other medical conditions. He did not take any medications and denied the use of tobacco or illicit drugs. He reported consuming one alcoholic beverage per week.

On examination, the patient was alert, afebrile, and mildly hypotensive. His abdomen was mildly distended. On palpation, diffuse tenderness and guarding were present. The bowel sounds were decreased. The rest of the physical exam was otherwise unremarkable. Laboratory studies showed a white blood cell count of 18.4 K/mm^3^ (normal: 3.7 - 10.5 K/mm^3^) and hemoglobin of 18.2 g/dL (normal: 13.2 - 17.7 g/dL). The basic metabolic panel, liver function tests, and lactic acid were unremarkable.

**Figure 1:**
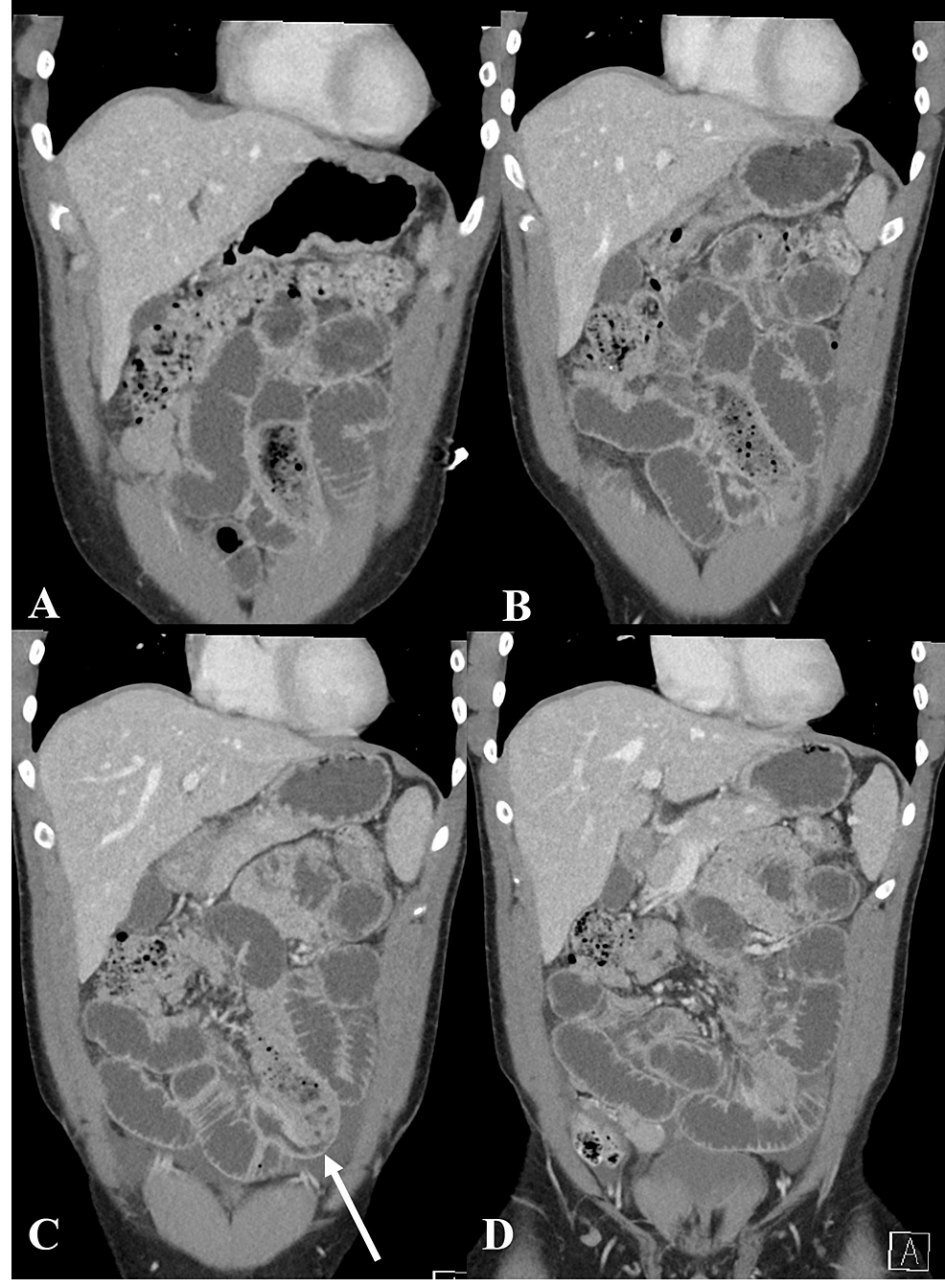
Dilated small bowel loops and transition point of the obstruction Contrast-enhanced CT of the abdomen and pelvis in coronal reformation from anterior to posterior (A-D) shows multiple dilated small bowel loops with fecalization of the contents in the left lower quadrant, and a transition point (white arrow).

**Figure 2:**
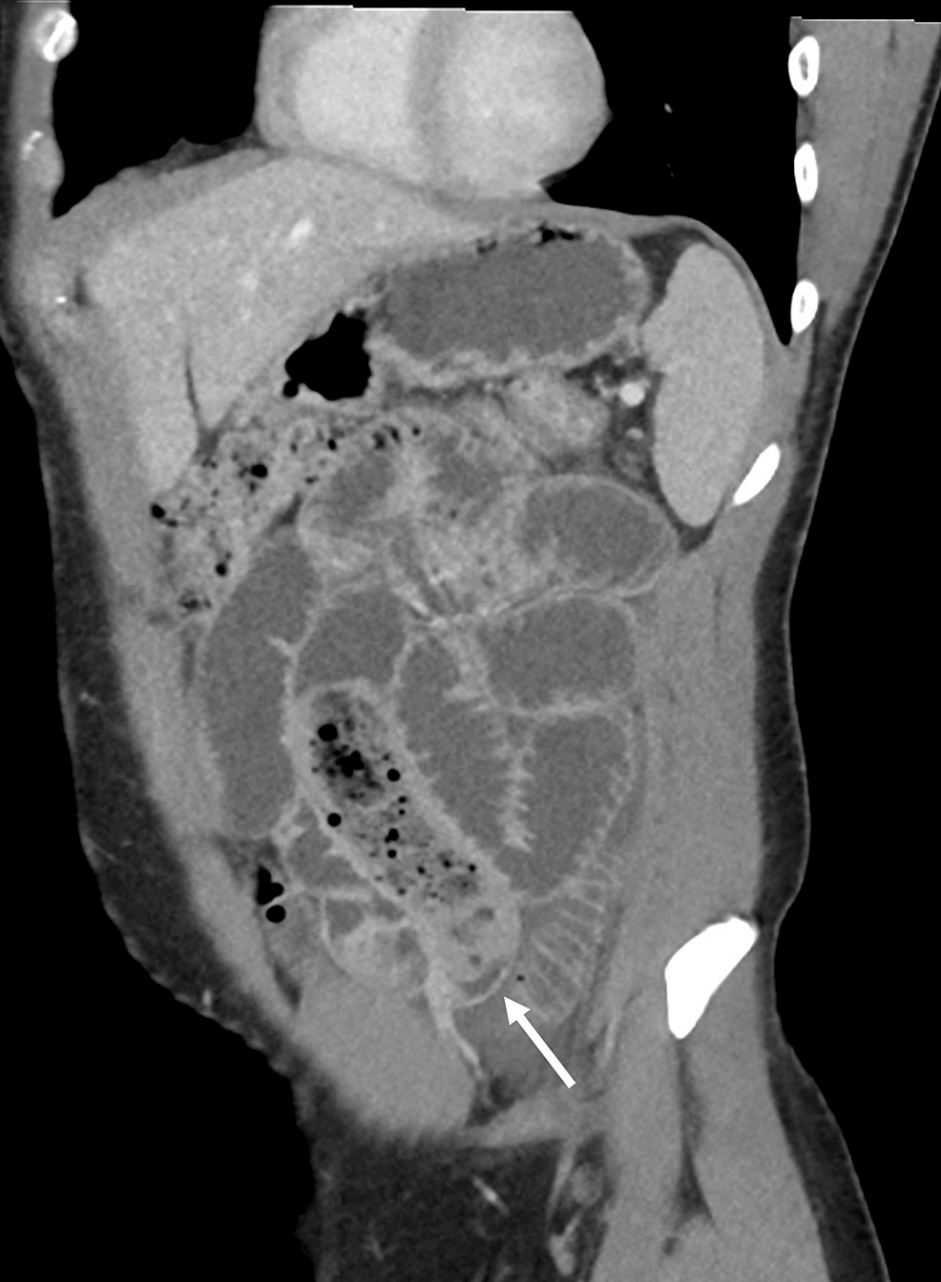
Transition point of the small bowel obstruction. Oblique coronal reconstruction shows fecalization of the small bowel caused by the banana-stuffed condom subsequently found at surgery. The solid white arrow shows the transition point.

During the evaluation, the patient became diaphoretic and hypotensive with systolic blood pressure in the 60s (mmHg). He then had two episodes of large-volume, non-bloody emesis. Administration of 1 liter of normal saline was initiated, and he had subsequent improvement of systolic blood pressure to the 100s (mmHg). A contrast-enhanced computed tomography (CT) of the abdomen and pelvis was notable for small bowel dilation up to 3.5 cm with fecalization, adjacent to a transition point in the left lower quadrant (Figures 1-2, Video 1). These findings were consistent with small bowel obstruction. A small amount of ascites was also present, concerning for bowel distress. Hence, the general surgery service was consulted.

**Video 1:** Contrast-enhanced axial abdominopelvic CT A cine clip of contrast-enhanced axial CT abdomen and pelvis in craniocaudal direction demonstrates multiple distended loops of bowel, small volume ascites, and fecalization of contents in the left lower quadrant.

**Figure 3:**
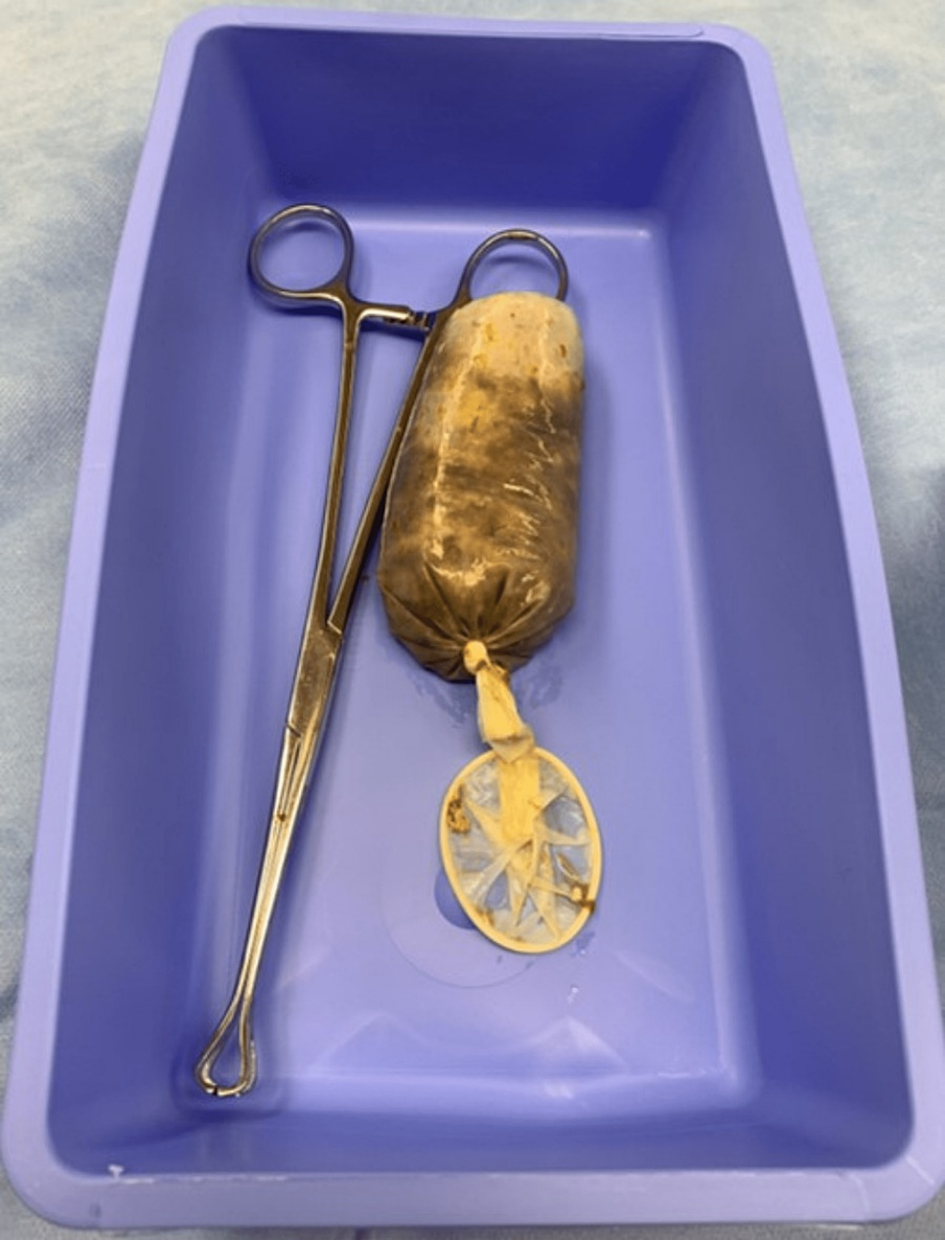
Banana in a condom Photograph of the banana-filled condom removed intraoperatively

Given the severity of the obstruction and concern for bowel distress, an exploratory laparotomy was indicated over expectant management. The patient was started on antibiotic prophylaxis with cefazolin and metronidazole and was placed under general anesthesia. Upon entering the peritoneum, small-volume ascites was visualized. Samples were obtained and cultured, which later grew Cutibacterium acnes. Examination of the small bowel identified high-grade obstruction in the jejunum, where an obvious foreign body was palpable, with an acute transition point. A 1-cm enterotomy was made proximal to the foreign body and the foreign body was removed intact (Figure 3). The enterotomy was closed transversely. Re-examination of the small bowel from the ligament of Treitz to the cecum did not identify any other foreign body or masses. The abdomen was irrigated, followed by closure by layers. The patient tolerated the procedure well and his postoperative recovery was uneventful. He was able to tolerate oral intake and had a return of bowel function. He was discharged on postoperative Day 3 without complications.

Two weeks after the operation, he was tolerating a low-fiber diet without nausea or vomiting. He had a return of normal bowel movements, and his pain was well-controlled. At six months, he continued to endorse normal bowel patterns and diet. He was able to slowly resume his active lifestyle and did not have major concerns.

## Discussion

Previous reports of condom ingestion have largely been related to the transportation (body packing) or concealment (body stuffing) of illicit drugs [3,4]. Most cases are asymptomatic and can be managed conservatively [5-7]. However, there are several reports of surgical indications in asymptomatic patients when spontaneous elimination fails, due to the risk of condom rupture associated with prolonged retention in the stomach [8,9]. Serious complications, such as drug toxicity, may occur following the rupture of the condom [10,11]. Chest pain, hypertension, tachycardia, and arrhythmias are often seen in cocaine intoxication, whereas heroin intoxication may produce sedation and respiratory depression.

Intestinal obstruction is another serious complication resulting from body packing or stuffing described in several reports [9,12,13]. An even more unusual cause of gastrointestinal obstruction is the ingestion of condoms filled with substances other than drugs. Few reports of ingested, fluid-filled condoms causing small bowel obstruction have been described. In one case, a patient reported a sensation of swallowing marbles while participating in a beer-drinking competition [14]. After failing to improve with conservative management, a laparotomy with subsequent enterotomy was performed to remove two 4-cm, fluid-filled condoms [14]. Another case describes a similar surgery performed to remove a fluid-filled condom, swallowed as part of a YouTube challenge, that led to the obstruction of the distal jejunum [15]. Others have reported a less invasive technique of image-guided transcutaneous tapping of fluid-filled condoms when easily accessible. Tapping under ultrasound guidance was performed on one patient who was admitted for swallowing a water-filled condom [16]. Another case describes a patient who swallowed a beer-filled condom while at Munich Octoberfest; CT-guided transabdominal tapping was performed after unsuccessful efforts to remove the condom by upper endoscopy [17].

A literature search on condom-related intestinal obstructions yielded numerous reports of cases wherein the condom was filled with drugs; few reports, however, involved fluid-filled condoms. To the best of our knowledge, the present case appears to be the first case in which a condom containing a banana was swallowed. Drug toxicity associated with condom rupture was, therefore, not a concern in our case as is seen in the setting of body packing or stuffing. Our patient underwent laparotomy and enterotomy as transcutaneous treatment was not an option regardless of accessibility given the solid consistency of a banana as compared to fluids.

## Conclusions

Condom ingestion rarely causes intestinal obstruction and is, therefore, treated expectantly. Conservative management commonly includes intravenous fluids, nasogastric suction, oral laxatives, or enemas. Clinical judgment based on history, symptoms, and radiographic signs should be used to determine indications for surgical management in cases of swallowed condoms containing unusual substances such as a banana. Early surgical intervention should be considered in patients with clinical and radiographic signs of high-grade small bowel obstruction. Moreover, the type of ingested foreign body causing the obstruction should be considered in the assessment of the indicated intervention.
